# Optimal 2-[^18^F]fluoro-2-deoxy-d-galactose PET/CT protocol for detection of hepatocellular carcinoma

**DOI:** 10.1186/s13550-016-0206-7

**Published:** 2016-06-24

**Authors:** Jacob Horsager, Kirstine Bak-Fredslund, Lars Peter Larsen, Gerda Elisabeth Villadsen, Trond Velde Bogsrud, Michael Sørensen

**Affiliations:** Department of Nuclear Medicine & PET Centre, Aarhus University Hospital, DK8000 Aarhus, Denmark; Department of Radiology, Aarhus University Hospital, DK8000 Aarhus, Denmark; Department of Hepatology & Gastroenterology, Aarhus University Hospital, Noerrebrogade 44 bldg. 7, DK8000 Aarhus C, Denmark; Department of Radiology and Nuclear Medicine, Oslo University Hospital, Oslo, Norway

**Keywords:** Hepatocellular carcinoma, Galactose, Dynamic PET, Static PET, Metabolic clearance

## Abstract

**Background:**

Positron emission tomography (PET) with the liver-specific galactose tracer 2-[^18^F]fluoro-2-deoxy-d-galactose (^18^F-FDGal) may improve diagnosis of hepatocellular carcinoma (HCC). The aim of this study was to test which of three different ^18^F-FDGal PET protocols gives the highest tumour-to-background (T/B) ratio on PET images and thus better detection of HCC tumours.

**Methods:**

Ten patients with a total of 15 hepatic HCC tumours were enrolled prior to treatment. An experienced radiologist defined volumes of interest (VOIs) encircling HCC tumours on contrast-enhanced CT (ce-CT) images. Three PET/CT protocols were conducted following an intravenous ^18^F-FDGal injection: (i) a 20-min dynamic PET/CT of the liver (to generate a 3D metabolic image), (ii) a traditional static whole-body PET/CT after 1 h, and (iii) a late static whole-body PET/CT after 2 or 3 h. PET images from each PET/CT protocol were fused with ce-CT images, and the average standardized uptake values (SUV) in tumour and background liver tissue were used to calculate (T/B) ratios. Furthermore, T_peak_/B ratios were calculated using the five hottest voxels in all *hot* tumours. The ratios for the three different PET protocols were compared.

**Results:**

For the individual tumours, there was no significant difference in the T/B ratio between the three PET protocols. The metabolic image yielded higher T_peak_/B ratios than the two static images, but it was easier to identify tumours on the static images. One extrahepatic metastasis was detected.

**Conclusions:**

Neither metabolic images nor static whole-body images acquired 2 or 3 h after ^18^F-FDGal injection offered an advantage to traditional whole-body PET/CT images acquired after 1 h for detection of HCC.

## Background

Hepatocellular carcinoma (HCC) is the most common primary liver cancer and the second most frequent cause of cancer-related death worldwide [1]. In more than 90 % of the cases, the development of HCC is associated with a known risk factor such as liver cirrhosis or chronic viral hepatitis [2]. The European Association for the Study of the Liver (EASL) recommends diagnosis of HCC be based on multiphase contrast-enhanced (ce-) CT and/or MRI with specific radiological hallmarks (arterial hypervascularity and venous/delayed washout). Two imaging protocols are recommended for small (<1 cm) tumours, and biopsy is recommended in non-cirrhotic cases and when radiology is inconclusive [3]. The challenge is to identify small tumours, which are potentially curable [3].

During malignant transformation, a tumour might change metabolism before morphological changes become visible on CT or MRI. For that reason, positron emission tomography with integrated computed tomography (PET/CT) is widely used for diagnosis and staging of a broad range of cancer types. The glucose analogue 2-[^18^F]fluoro-2-deoxy-d-glucose (^18^F-FDG) is the most commonly used PET tracer for detecting malignancies, but for HCC, the diagnostic sensitivity of ^18^F-FDG PET/CT is only 50–70 % [4–7].

Galactose is almost exclusively metabolized in hepatocytes, and a proof-of-concept PET/CT study of 39 patients with the galactose analogue 2-[^18^F]fluoro-2-deoxy-d-galactose (^18^F-FDGal) showed promising results for detection of HCC [8]. For hepatic lesions, the sensitivity of ^18^F-FDGal PET/CT was similar to that of ce-CT, and ^18^F-FDGal PET/CT detected previously unknown extrahepatic disease in eight patients [8]. The tumour-to-background (T/B) ratio was however relatively low for hepatic lesions, and we hypothetized that the ratio between tumour and background could be enhanced by delayed PET/CT acquisition 2 or 3 h after tracer administration as it has been suggested for ^18^F-FDG [9]. In addition, the so-called metabolic images of the hepatic clearance of ^18^F-FDGal created using a dynamic ^18^F-FDGal PET/CT protocol can be used to quantify regional hepatic metabolic function in vivo [10, 11]. Metabolic images might have an advantage to static imaging as it was shown for ^18^F-FDG PET of cholangiocarcinoma [12] because they depict differences in metabolic capacity, i.e. enzymatic activity.

The aim of the present study was to determine whether a metabolic image (generated from a 20-min dynamic PET/CT) or a late static PET/CT 2 or 3 h after tracer injection might provide better tumour detection than a traditional static PET/CT after 1 h.

## Methods

Ten patients with HCC diagnosed according to the EASL-EORTC guidelines [3] were enrolled in the study. All tumours were visible on multiphase ce-CT, and the diagnosis was confirmed by histology in seven patients. The ce-CT was performed on average 22 days before the present study (range, 4–46 days) and according to guidelines [3]. Patients were enrolled prior to treatment. Three patients had previously been treated for HCC; two had been treated with transarterial chemoembolization and one had undergone hepatic resection, but all three subjects were presenting with relapse now and enrolled before treatment of their recurrent tumours.

The study was approved by The Central Denmark Region Committees on Health Research Ethics and conducted in accordance with the Helsinki Declaration. Informed consent was obtained from all individual participants included in the study.

### ce-CT protocol

The CT images of the liver were obtained with a 64-detector CT scanner (Brilliance™, Philips Healthcare, Best, Netherlands). The following CT parameters were used: 64 × 0.625 mm collimation, section thickness 2.0 mm, increment of 1.0, 120–140 kVp, and 200–250 mAs. A bolus of minimum 85 mL to a maximum of 153 mL (1.7 mL/kg total body weight) non-ionic contrast agent (Visipaque™, 320 mg I/mL; GE Healthcare, Oslo, Norway) was administered to all patients at a rate of 4.0 mL/s. A bolus-tracking technique was used to compensate for differences in cardiac output. The trigger region of interest (ROI) was placed in the thoracic aorta, and when it exceeded 150 HU, the liver was scanned with a delay of 15 s (arterial phase) and 50 s (portal venous phase).

### ^18^F-FDGal PET/CT protocol

All subjects were studied in supine position using the same PET/CT camera, a 64-slice Siemens Biograph Truepoint PET/CT camera (Siemens AG, Erlangen, Germany). Patients fasted for at least 6 h before the study but were encouraged to drink water. Nine subjects had an Artflon catheter (Artflon, Ohmeda, Swindon, UK) placed percutaneously in a radial artery for blood sampling during the dynamic PET protocol.

#### Dynamic PET/CT

Before the dynamic PET/CT, a topogram was performed for optimal positioning of the liver within the 216-mm transaxial field of view of the PET camera followed by a low-dose CT scan (50 effective mAs with CARE Dose4D, 120 kV, pitch 0.8, slice thickness 5 mm) for attenuation correction of PET data. At time = 0, the dynamic PET scan was started (list mode) and a bolus of 100 MBq ^18^F-FDGal in 10 mL saline was administered intravenously in a cubital vein over the time course of 15–20 s. ^18^F-FDGal was produced in our own radiochemistry laboratory (radiochemical purity ≥97 %) [13]. Arterial blood samples (0.5 mL) were collected manually at times 18 × 5 s, 1 × 10 s, 4 × 20 s, 2 × 30 s, 2 × 1.5 min, and 6 × 2 min, total 19 min (the last blood sample at the midpoint of the last reconstructed time frame), for determination of blood concentrations of ^18^F-FDGal (kBq/mL blood) using a well counter (Packard Instruments, Meriden, CT, USA) and corrected for radioactivity decay back to time = 0. Time course of the blood concentration of ^18^F-FDGal in the patient without Artflon (patient #1) was obtained from a volume of interest in the posterior part of the aorta (aorta-VOI) [14].

#### Static PET/CT

One hour after tracer administration, a standard whole-body PET/CT was performed from the mid-thigh to the cranial vertex with an acquisition time of 3 min/bed position (5–6 bed positions) and low-dose CT scan (50 effective mAs with CARE Dose4D, 120 kV, pitch 0.8, slice thickness 5 mm) for attenuation correction of PET data. Two (patients #1–4) or three (patients #5–10) hours after tracer administration, the static PET/CT protocol was repeated with an acquisition time of 4 min per bed position for the protocol after 2 h and 5 min per bed position for the protocol after 3 h. The acquisition time after 2 and 3 h was increased to account for radioactive decay of the injected ^18^F-FDGal.

#### Reconstruction of data

The dynamic PET data were reconstructed with resolution modelling, 336 × 336 matrix, voxel size 2 × 2 × 2 mm^3^, 4 iterations, 21 subsets, separate prompts/randoms, and a 2.0-mm FWHM Gaussian post reconstruction filter. Measurements were corrected for radioactivity decay back to *t* = 0. Time-frame structure was 20 × 5 s, 1 × 10 s, 3 × 20 s, 1 × 30 s, 1 × 40 s, 2 × 1 min, and 7 × 2 min, total 20 min. From the dynamic PET data, metabolic images were generated using the commercially available PMOD software (Pixelwise Modelling Tool, PMOD Technologies). Briefly, Gjedde-Patlak kinetic analysis [15, 16] was applied to each voxel in the reconstructed PET images using the time course of radioactivity concentration in arterial blood as input function (aorta-VOI in patient #1) and the time course of radioactivity concentration in each voxel (liver tissue) as output function. The procedure yields 3D metabolic images of the regional hepatic blood clearance of ^18^F-FDGal (*K*, mL blood/mL tissue/min) [10, 11].

The static PET data were reconstructed using resolution modelling, 336 × 336 matrix, voxel size 2 × 2 × 2 mm^3^, 3 iterations, 21 subsets, separate prompts/randoms, and a 3.0-mm FWHM Gaussian post reconstruction filter. Standardized uptake values (SUV) of ^18^F-FDGal were calculated by dividing the tissue radioactivity concentration by the injected dose and body weight.

### Data analysis

**Fig. 1:**
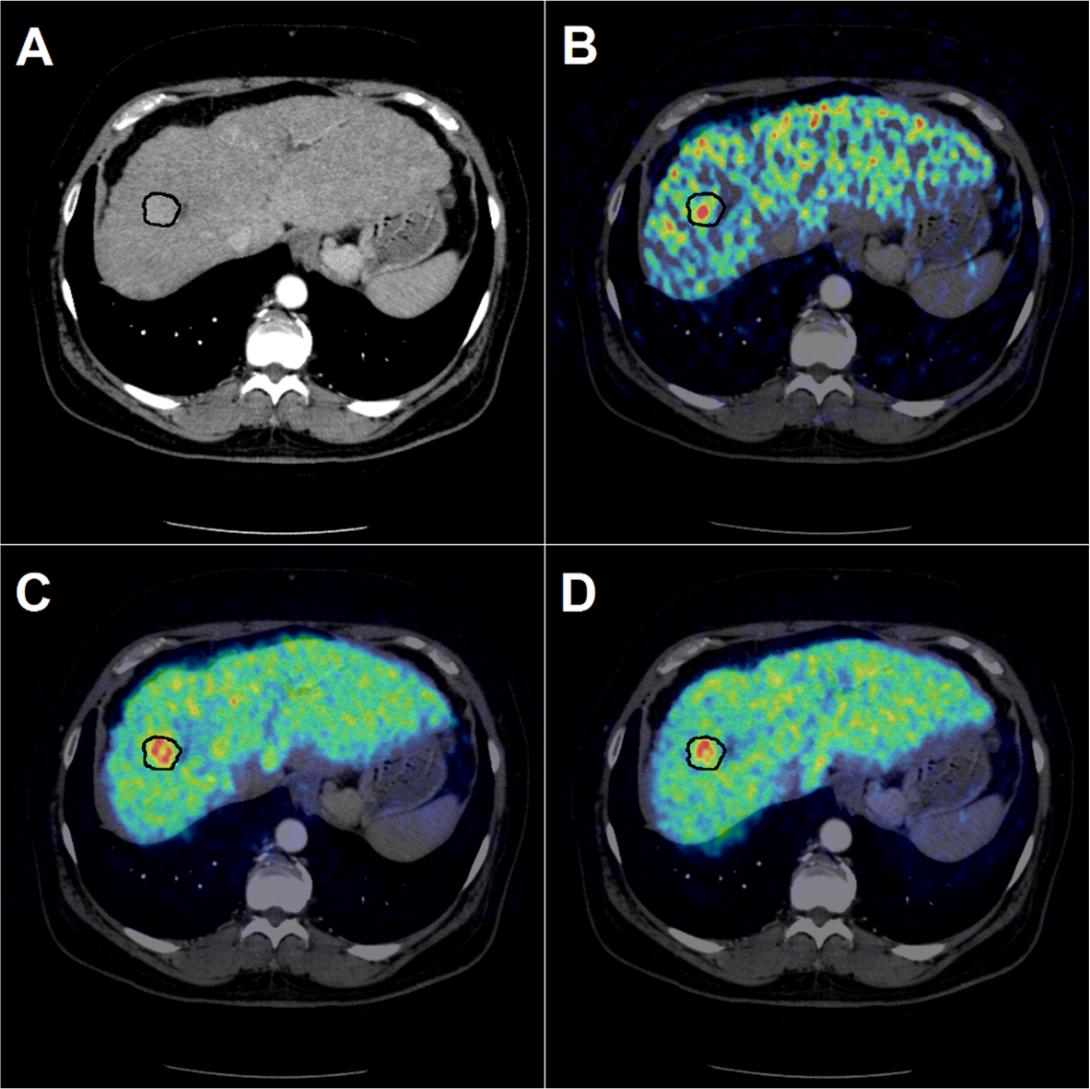
Contrast-enhanced CT image (arterial phase) with VOI-definition around the hypervascular HCC tumour (**a**) fused with the metabolic PET image (**b**), with the 1-h static image (**c**), and with the 2-h static image (**d**)

An experienced radiologist (LPL) identified HCC tumours on the diagnostic ce-CT, and volumes-of-interest (VOIs) were defined encircling all visible tumours (tumour-VOI). Using the fusion tool in PMOD, the reconstructed PET images were fused with the diagnostic ce-CT and the VOI was moved in 3D to ensure the best possible match (Fig. 1).

A VOI in background liver tissue was defined in an area without tumours and large blood vessels, approximately 1 cm from the edge of the liver (average volume was 39 mL liver tissue; range, 10–83 mL liver tissue), and used as background-VOI. The average values (*K* from metabolic images and SUV from static images) were obtained in the tumour-VOI and the background-VOI to calculate a T/B ratio. Tumours with a ratio <1 were defined as *cold*, and tumours with a ratio >1 were defined as *hot*. Furthermore, a T_peak_/B ratio was calculated using the average values of the five voxels with the highest values in the *hot* tumours (*K* from metabolic images and SUV from static images) divided by the average value in background liver tissue. The images from the static whole-body PET/CT were carefully inspected for lesions with high ^18^F-FDGal accumulation outside the liver (extrahepatic disease).

### Statistics

Volumes of the tumour-VOI in *hot* vs. *cold* lesions were compared by the Mann-Whitney *U* test (non-normal distribution). T/B ratios from parametric images (*K*) and late static scan (SUV) were compared to T/B ratios from the static PET scan after 1 h using a paired *t* test. The paired *t* test was also used to compare T_peak_/B ratios from parametric and late static PET scan against the PET scan after 1 h. A *P* value <0.05 was considered to indicate a statistically significant difference.

## Results

This study included 10 patients with a total of 15 hepatic lesions; patient characteristics are shown in Table 1. Nine patients had cirrhosis, and the aetiology was alcohol in six patients and hepatitis C virus (HCV) in three patients.

**Table 1:** Patient characteristics

Patient ID	Age	Sex	Number of HCC tumours	Histology confirmed	Cirrhosis	Child-Pugh class [17]	Aetiology	aFP (ng/mL plasma)	Time from ce-CT to PET (days)
1	53	F	1	+	+	C	HCV	3	46
2	71	M	2	+	+	A	Alcohol	295,800	46
3	65	M	1	+	+	A	Alcohol	11	9
4	62	M	2	−	+	C	Alcohol	55	4
5	70	F	1^a^	+	−	N/A	−	4	13
6	69	M	1	+	+	B	HCV	17,102	13
7	61	F	1	+	+	A	HCV	298	15
8	67	M	1	+	+	B	Alcohol	N/A	35
9	64	M	4	−	+	A	Alcohol	38	11
10	59	M	1	−	+	A	Alcohol	4	25

*F* female, *M* male, *HCC* hepatocellular carcinoma, *HCV* hepatitis C virus, *aFP* alpha-fetoprotein, *ce-CT* contrast-enhanced CT, *N/A* not applicable

^a^This patient had multiple tumours, but only one was isolated for VOI-definition on ce-CT scan

**Fig. 2:**
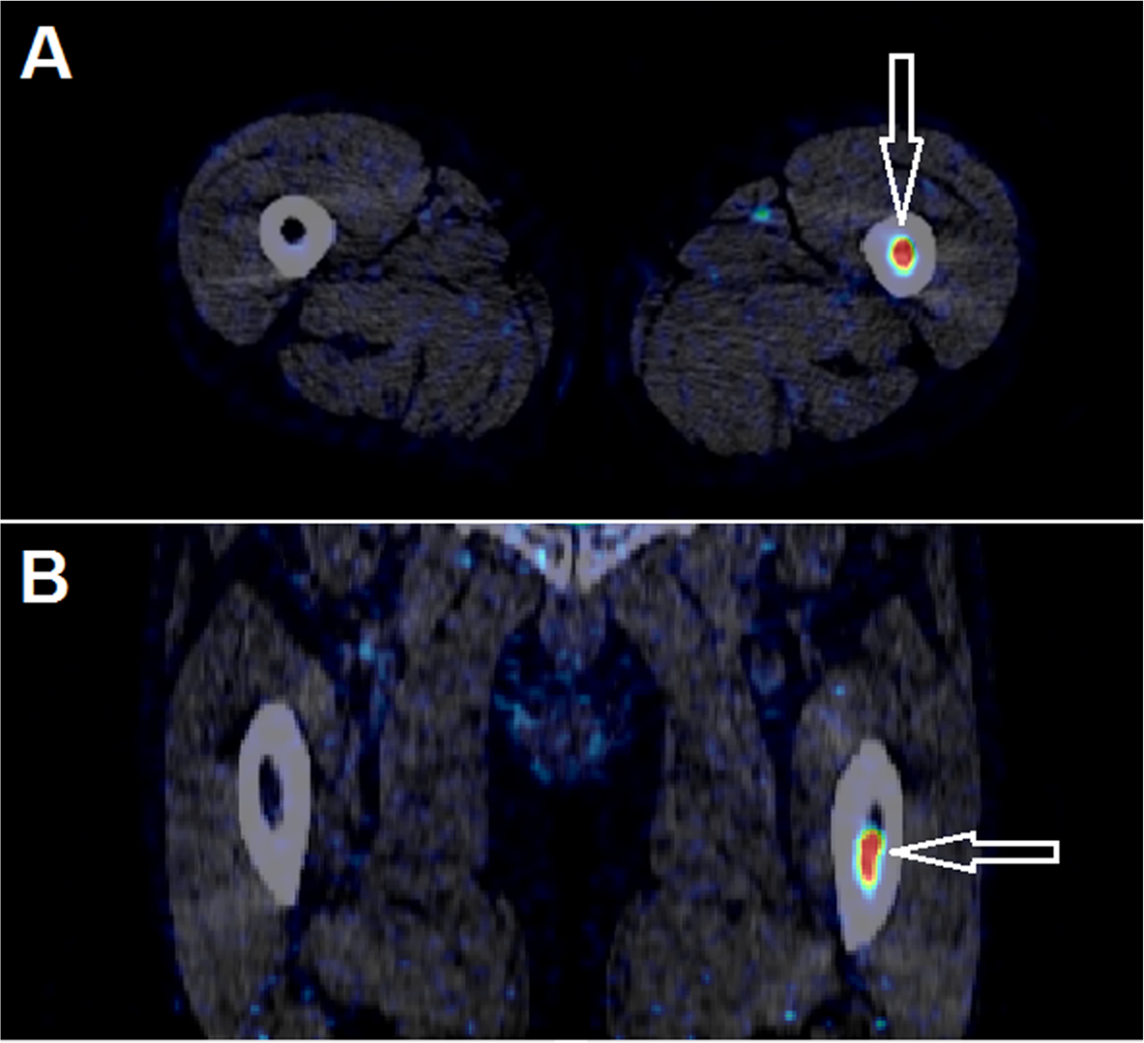
*Arrows* show a metastasis in the left femoral bone of patient #6 detected on a static ^18^F-FDGal PET image (visible on both static images; here, the 1-h static image is shown). **a** Transaxial slice. **b** Coronal slice

Of the 15 hepatic lesions, eight were defined as *hot* and seven as *cold*. Twelve/fifteen tumours found on ce-CT were identified on ^18^F-FDGal PET/CT. Six tumours were easier to identify on the static images, one was easier to identify on the metabolic image, and five tumours were identified equally well using all three PET/CT protocols. Three of the ce-CT-diagnosed tumours could not be identified on PET images (tumours 2B, 9A, and 9D in Table 2). As seen in Table 2, tumour 2B had the highest T/B ratio among the *cold* tumours, i.e. closest to unity, and tumours 9A and 9D had the lowest T/B ratios among the *hot* tumours, i.e. closest to unity. The calculated ratios were thus in agreement with the visual impression of these three tumours as they differed least from the background liver tissue. One previously unknown extrahepatic metastasis in the left femoral bone was detected in patient #6, and it was visible on PET images acquired after 1 and 3 h (Fig. 2).

**Table 2:** Tumour characteristics

Patient ID	Tumour ID	Tumour volume (mL)	T/B ratio metabolic image	T/B ratio static; 1 h	T/B ratio static; 2 or 3 h	T_peak_/B ratio metabolic image	T_peak_/B ratio static; 1 h	T_peak_/B ratio static; 2 or 3 h
1	1A (cold)	8.6	0.44	0.51	0.49	–	–	–
2	2A (cold)	1548	0.56	0.52	0.55	–	–	–
	2B (cold)	39	0.86	0.89	0.90	–	–	–
3	3A (hot)	10	1.42	1.49	1.38	2.72	2.39	2.43
4	4A (hot)	7.8	1.61	1.56	1.46	4.94	2.56	3.13
	4B (hot)	0.92	1.69	1.76	1.93	3.84	3.21	3.74
5	5A (cold)	11	n.d.	0.69	0.73	–	–	–
6	6A (cold)	492	0.58	0.59	0.64	–	–	–
7	7A (cold)	21	0.60	0.82	0.86	–	–	–
8	8A (hot)	17	1.05	1.12	1.14	2.60	2.06	2.13
9	9A (hot)	0.43	0.97	1.19	1.10	1.59	1.48	1.30
	9B (hot)	5.0	1.29	1.26	1.24	2.74	2.06	1.98
	9C (hot)	13	1.39	1.33	1.31	4.73	3.38	3.36
	9D (hot)	0.58	1.19	1.18	1.16	1.80	1.47	1.32
10	10A (cold)	311	0.66	0.61	0.57	–	–	–
Average	(cold)		0.62	0.66	0.68	–	–	–
Average	(hot)		1.33	1.36	1.34	3.12*	2.33	2.42

T/B ratio: ratio between the average value in tumour to the average value in background liver tissue. T_peak_/B ratio: ratio between the average value of the five hottest voxels in tumour to the average value in background liver tissue. The late static scan was performed after 2 h in patients #1–4 and after 3 h in patients #5–10

*n.d.* not determined because of major respiratory artefacts

*P ≤ 0.05 when compared to T_peak_/B ratio static 1 h

The three patients with HCV all had *cold* tumours, and all *hot* tumours were found in patients with alcohol-induced cirrhosis. One patient had multiple tumours in a non-cirrhotic liver (patient #5). The 12 tumours visible on PET had the same appearance on all three tested methods, i.e. a *hot* tumour on the metabolic image was also *hot* on the two static images. The three unidentified tumours (by PET) where considered *hot/cold* depending on their ratios. In tumour 9A, the ratio from the metabolic image was 0.97, i.e. *cold*, but >1 in the two static images, i.e. *hot* (Table 2). This tumour was categorized as *hot* as the two static images were considered the most reliable.

The median tumour volume, based on ce-CT, was 7 mL (range, 0.4–17 mL) for *hot* tumours and 39 mL (range, 9–1550 mL) for *cold* tumours (*U* = 5, *P* < 0.01).

Table 2 shows the characteristics for each hepatic lesion for each of the three PET/CT protocols. The average T/B ratio for *cold* tumours was 0.62 in the metabolic images, 0.66 in the 1-h static images, and 0.68 in the 2- or 3-h static images (*P* > 0.3 for all). The average T/B ratios for *hot* tumours were 1.33, 1.36, and 1.34, respectively (*P* > 0.3 for all; Table 2). Tumours 4B, 7A, and 9A did however have a higher or lower observed value in one protocol compared to the two others (Table 2); 4B had a higher ratio in the 2-h static image, and both 7A and 9A had a lower value in the metabolic image compared to the two other PET/CT protocols. However, the deviations were not systematic since the other tumours did not follow this pattern.

**Fig. 3:**
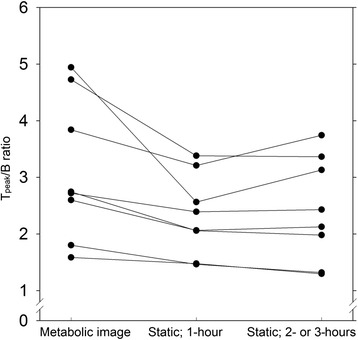
T_peak_/B ratio for all hot tumours (average of the five voxels with the highest value in tumour divided by the average background value). Individual tumours are connected with a *line*

T_peak_/B ratios for the tumours are given in Table 2. Metabolic images systematically yielded higher T_peak_/B ratios than the two static images (Fig. 3) with a mean T_peak_/B ratio of 3.12 vs. 2.33 for the 1-h static PET scan (*P* < 0.05). There was no statistically significant difference between the T_peak_/B ratios from the two static protocols (*P* > 0.3), but the two ratios were higher in the 2- or 3-h static protocol than in the 1-h static protocol (tumours 4A and 4B, Table 2).

Since the analysis was based on HCC lesions visible on ce-CT and co-registered with PET images, we did not observe any interobserver differences in the results (data not shown).

## Discussion

The main result from the study is that there were no significant differences in the T/B ratio for intrahepatic HCC lesions when comparing the three tested ^18^F-FDGal PET/CT protocols, i.e. metabolic images, static images after 1 h, and static images after 2 or 3 h after intravenous injection of ^18^F-FDGal. The static PET images detected previously unknown extrahepatic disease in one patient, which underlines the importance of performing whole-body PET and not just PET of the liver. The extrahepatic metastasis was identified equally well on the two static image protocols.

The study was conducted in patients with known HCC tumours because the aim was to evaluate the differences between three ^18^F-FDGal PET imaging protocols and not to test the diagnostic performance of ^18^F-FDGal PET vs. conventional morphologic imaging modalities (ultrasound, ce-CT, and MRI). The number of patients may be relatively small but with a strong, paired design. With the results from the present study, a sample size of several thousands would have been necessary to show a statistical difference which means that for all practical purposes, there is no difference. Based on this, we did not find it ethical to include more patients.

Interpretation of ^18^F-FDGal PET/CT images is challenged by the very high uptake in normal liver tissue and heterogeneous uptake in cirrhotic patients. To identify HCC tumours, tracer uptake must be either significantly higher or lower in tumour compared to background liver tissue. The intrahepatic variation in tracer uptake is more pronounced in the metabolic images than in the smoother static image because of more image noise and probably because the metabolic images provide clearance values whereas tracer in blood and non-metabolized tracer in hepatocytes contribute to the radioactivity concentration in the static images. This might be the reason why six intrahepatic HCC lesions were easier to identify on the static images and only one was easier to identify on the metabolic image in spite of the fact that statistically, the metabolic images yielded higher T_peak_/B ratios than the static images. Moreover, this analysis was only possible to conduct in 8/15 tumours because the remaining tumours were classified as *cold* and metabolic images are also only obtained from the liver whereas static images can be performed as whole-body scans. In this study, a previously unknown metastasis was identified in the left femoral bone in patient #6 (Fig. 2). A proof-of-concept study evaluating 39 patients with HCC with a static whole-body ^18^F-FDGal PET/CT found extrahepatic disease in nine patients of which eight were novel findings [8]. A whole-body static PET/CT is thus essential for detecting extrahepatic metastases in patients with HCC.

The diagnostic ce-CT and the PET images were fused manually (the PET images had been attenuation-corrected by the low-dose CT) using the PMOD fusion tool. Since the two datasets were not obtained on the same day and the ce-CT was acquired during inspiratory breath-hold while PET/CT was acquired during breathing, the two image series did not fit perfectly but a close correlation around the tumour area was prioritized. Three tumours (2B, 9A, and 9D) were not visible on PET, and the correlation between tumour-VOI and the actual tumour area on PET might thus be poor. However, since the tumours could not be identified on any of the three PET images, this probably did not affect our results.

In the present study, a *cold* lesion was defined as a ce-CT-defined tumour with a low tracer uptake on the ^18^F-FDGal images compared to background liver tissue on PET fused with ce-CT. However, tumour 2A had a large cold area defining the tumour as *cold*, but *hot* tissue was also present at the rim of the tumour. Both areas were encircled as tumour on ce-CT, and one might argue that this tumour was both *hot* and *cold. Cold* areas in tumours might be caused by central necrosis, but the average SUV for *cold* tumours were approximately 14, which indicates that even a *cold* tumour has a relative high uptake of ^18^F-FDGal compared to other tissues. In the present study, *cold* tumours were statistically larger than *hot* tumours, median 39 vs. 6 mL (*P* < 0.01). Furthermore, the *hot* tumours had, in general, a regular spherical shape (Fig. 1), whereas the larger *cold* tumours were more irregular in shape with less clearly defined borders. The *cold* tumours might thus be more aggressive and dedifferentiated, and ^18^F-FDGal PET/CT may provide prognostic value. However, since large tumours are rather easy to identify on ce-CT, diagnostic improvement must be focused on small tumours. It is likely that ^18^F-FDGal PET/CT will be more useful in finding small, *hot* lesions in patients with alcohol cirrhosis than in patients with chronic HCV, but it is beyond the scope of the present study to answer that question which should be addressed in a large prospective study.

## Conclusions

Static whole-body PET performed 1 h after injection of 100 MBq ^18^F-FDGal is the recommended protocol for both intra- and extrahepatic HCC lesions since neither metabolic nor late static whole-body PET images offered any advantages.
